# The importance of anaemia in diagnosing colorectal cancer: a case–control study using electronic primary care records

**DOI:** 10.1038/sj.bjc.6604165

**Published:** 2008-01-22

**Authors:** W Hamilton, R Lancashire, D Sharp, T J Peters, K K Cheng, T Marshall

**Affiliations:** 1 Academic Unit of Primary Health Care, Department of Community Based Medicine, University of Bristol, 25 Belgrave Road, Bristol BS8 2AA, UK; 2 Department of Public Health and Epidemiology, University of Birmingham, Edgbaston, Birmingham B15 2TT, UK

**Keywords:** anaemia, colorectal cancer, diagnosis, primary health care

## Abstract

Although anaemia is recognised as a feature of colorectal cancer, the precise risk is unknown. We performed a case–control study using electronic primary care records from the Health Improvement Network database, UK. A total of 6442 patients had a diagnosis of colorectal cancer, and were matched to 45 066 controls on age, sex, and practice. We calculated likelihood ratios and positive predictive values for colorectal cancer in both sexes across 1 g dl^−1^ haemoglobin and 10-year age bands, and examined the features of iron deficiency.In men, 178 (5.2%) of 3421 cases and 47 (0.2%) of 23 928 controls had a haemoglobin <9.0 g dl^−1^, giving a likelihood ratio (95% confidence interval) of 27 (19, 36). In women, the corresponding figures were 227 (7.5%) of 3021 cases and 58 (0.3%) of 21 138 controls, a likelihood ratio of 41 (30, 61). Positive predictive values increased with age and for each 1 g dl^−1^ reduction in haemoglobin. The risk of cancer for current referral guidance was quantified. For men over 60 years with a haemoglobin <11 g dl^−1^ and features of iron deficiency, the positive predictive value was 13.3% (9.7, 18) and for women with a haemoglobin <10 g dl^−1^ and iron deficiency, the positive predictive value was 7.7% (5.7, 11). Current guidance for urgent investigation of anaemia misses some patients with a moderate risk of cancer, particularly men.

Iron-deficiency anaemia has long been recognised as a feature of colorectal cancer (Goodman and Irvin, 1993). It is present in 11–57% of cancers (Curless *et al*, 1994; Majumdar *et al*, 1999; Roncoroni *et al*, 1999; Young *et al*, 2000) and is particularly suggestive of caecal tumours (Dunne *et al*, 2002). Patients with anaemia as their presenting feature of cancer have worse staging (Gonzalez-Hermoso *et al*, 2004) and mortality (Stapley *et al*, 2006). Thus, referral of patients with iron-deficiency anaemia for investigation of possible gastrointestinal blood loss is recommended. The main source of referral advice is the Referral Guidelines for Suspected Cancer (NICE, 2005). These guidelines, updated in 2005 and sent to all UK general practitioners, suggest urgent referral for unexplained iron-deficiency anaemia, in a man of any age with a haemoglobin <11 g dl^−1^, or a nonmenstruating woman with a haemoglobin <10 g dl^−1^. Different advice comes from the British Society of Gastroenterology (BSG) guidelines, which suggests investigation of iron deficiency in men, and women over 50 or postmenopausal, once the haemoglobin value is below the laboratory's quoted normal range, but without suggesting a level of haemoglobin requiring urgent investigation (Goddard *et al*, 2005).

There is very little research evidence to guide the choice of a threshold haemoglobin warranting investigation. Only three studies have estimated the risk of colorectal cancer posed by iron-deficiency anaemia, and none for a specific threshold. The first study examined 695 patients referred for investigation under the BSG guidance and identified colorectal cancer in 6.3%. Cancer was more common in men, those over 50, and in those with a haemoglobin of 9.0 g dl^−1^ or below (James *et al*, 2005). The second examined 431 patients with iron-deficiency anaemia (haemoglobin below 12 g dl^−1^ in men and 11 g dl^−1^ in women, with microcytosis) and 32 (7.4%) colorectal cancers were identified (Logan *et al*, 2002). The degree of anaemia was not associated with the likelihood of cancer (Yates *et al*, 2004). Finally, a smaller study in a single general practice followed a cohort of 26 patients with iron-deficiency anaemia for 5 years, with two colorectal cancers being discovered (Stellon and Kenwright, 1997).

Less clear is the need for investigation of anaemia without iron deficiency, or for iron deficiency without anaemia. In iron deficiency without anaemia, two studies have estimated that the risk of cancer is around five times the background risk (Ioannou *et al*, 2002; Sawhney *et al*, 2007). Both these studies were based on small numbers of cancers, and were of patients already selected for investigation.

A third study examined the primary care records of 349 colorectal cancer patients and 1788 age- and sex-matched controls, and calculated positive predictive values (PPVs) of 0.97% for colorectal cancer for haemoglobin values in the range 10.0–12.9 g dl^−1^ and 2.3% for values below 10 g dl^−1^, but did not examine markers of iron deficiency (Hamilton *et al*, 2005). Conversely, patients with anaemia and a ferritin above 100 ng ml^−1^ have a risk of cancer little above those who are neither anaemic nor iron-deficient (Sawhney *et al*, 2007).

In the general population, anaemia is more common with increasing age. A large prevalence study was reported from the United States in 2004, with anaemia defined as a haemoglobin <13.0 g dl^−1^ in men and <12.0 g dl^−1^ in women (Guralnik *et al*, 2004). In the age group of 50–64 years, 4.4% of men and 6.6% of women were anaemic; the percentage with anaemia increased progressively with each age band up to 85+ years, when 26.1% of men and 20.1% of women were anaemic.

The incidence of colorectal cancer also rises with age: annual rates per 100 000 in 50- to 54-year olds are 44.7 in men and 33.1 in women; and 504 and 323, respectively, in those aged 85+ (Cancer Research UK, 2003). As both anaemia prevalence and colorectal cancer incidence rise with age, it is possible that the relative risk of colorectal cancer with anaemia is largely independent of age; this is a tacit assumption of current guidance. No study has been large enough to calculate the risk of colorectal cancer with anaemia for different ages, sexes, and haemoglobin values. This study aimed to address this evidence gap.

## METHODS

We studied data from The Health Improvement Network (THIN), which uploads electronic medical records from GP practices using the VISION computer system. The records contain patient characteristics, all consultations, diagnoses, and primary care investigations. The database has 2.2 million currently active patients in over 300 practices; 4.7 million patients when historical data are included. Laboratory results have been transmitted electronically to most practices from the year 2000.

### Identification of cases and controls

All patients with colorectal cancer were identified, who were aged 30 years or older, and diagnosed between January 2000 and July 2006. Up to seven controls were randomly selected for each case, using a computerised random numbers sequence. Controls were free from colorectal cancer and were matched for practice, sex, and age. Where possible controls were born in the same year as cases, if none were available within 1 year of the cases, the range was expanded to 2 years, continuing to a maximum of 5 years. All participants had at least 2 years of electronic records prior to the date of diagnosis of the case (the index date). The above stages of the data preparation were performed by THIN staff.

Haemoglobin results were taken in the year before the index dates were studied. If more than one haemoglobin measurement had been taken, the final value was used for analysis. Mean cell volume (MCV) and ferritin results for the same year were also collated. Microcytosis was defined as an MCV <80.0 fl, and a low ferritin as <20 ng ml^−1^.

### Analysis

Associations between anaemia, microcytosis, and low ferritin and colorectal cancer were estimated using multivariable conditional logistic regression. For the calculation of PPVs, haemoglobin values were stratified into six bands: below 9.0 and 1 g dl^−1^ bands thereafter, up to 13.0 g dl^−1^. Age was stratified into four bands: 30–59 (there were too few cancers in this band for meaningful subdivision) and 10-year bands thereafter, up to 80+ years. The sexes were analysed separately. For each age/haemoglobin/sex triad, we calculated the positive likelihood ratio with 95% confidence intervals from 2 × 2 tables. We used Bayes’ theorem (posterior odds=prior odds × likelihood ratio) to estimate PPVs (Knottnerus, 2002). The prior odds were derived from national incidence rates stratified by age and sex for 2003, in which an annual incidence of (for example) 5 per 1000 is the equivalent of 199 to 1 odds against having cancer diagnosed in the next year (Cancer Research UK, 2003). This was repeated for those patients with markers for iron deficiency.

As the number of cases was effectively predetermined, a power calculation, rather than a sample size calculation, was performed. For an estimated 5000 cases, there was >99% power to detect 5% having haemoglobin values in a particular band, compared with 1% among controls. These estimates were derived from one previous primary care study (Hamilton *et al*, 2005). Ethical approval was obtained from London MREC.

## RESULTS

Between 1 January 2000 and 31 July 2006, there were 6442 cases of colorectal cancer (3421 men, 3021 women) recorded on the THIN database; 45 066 matched controls were available. Of these, 3183 (49%) cases and 10 514 (23%) controls had a haemoglobin value measured in the year before the index date. Cases’ haemoglobin results were taken in a median (interquartile range) of 62 (28 122) days before the index date; equivalent figures for controls were 134 (59 235) days. Details of the cases and controls are shown in Table 1, and the haemoglobin results in Table 2.

Measurement of haemoglobin was more frequent in women; 6870 women (28.4%) had a haemoglobin result compared with 6827 men (25.0%); *P*<0.001, from a χ^2^ test. The positive likelihood ratios for each level of haemoglobin were relatively constant across the age bands.

An MCV result was available for 12 599 (92%) and a ferritin was recorded in 1548 (11%) of the 13 697 with a haemoglobin result. In cases, 764 (26%) of the 2951 MCV results showed microcytosis, and 353 (49%) of the 723 ferritin results were low. In controls, 210 (2.2%) of the 9648 MCV results showed microcytosis, and 129 (16%) of the 825 ferritin results were low.

**Figure 1:**
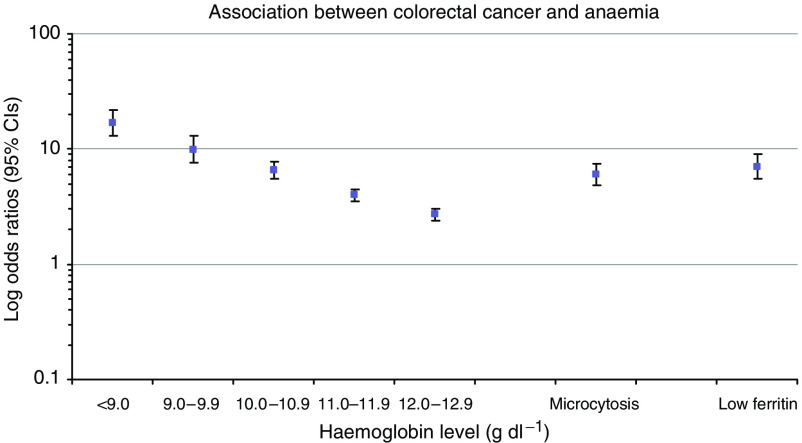
Semi-logarithmic plot of odds ratios for independent associations of colorectal cancer with different haemoglobin values and microcytosis and low ferritin results.

In a multivariable conditional logistic regression model, there were strong (*P*<0.001) independent associations between colorectal cancer and anaemia, microcytosis, and a low ferritin; these are shown in Figure 1. There was an antagonistic interaction between microcytosis and low ferritin (interaction OR 0.25 (0.15, 0.43)).

Positive predictive values for anaemia alone (irrespective of markers of iron deficiency) are shown for the two sexes in Tables 3 and 4. For the subgroup who had markers of iron deficiency, results are shown in Tables 5 and 6.

Positive predictive value for current recommendations was calculated for the those aged >60 years; men with markers of iron deficiency and a haemoglobin <11.0 g dl^−1^ had a PPV of 13.3% (CI 9.7, 18) and women with a haemoglobin <10.0 g dl^−1^ had 7.7% (5.7, 11). To allow comparison with the one previous primary care study, PPVs were calculated for two haemoglobin ranges, across all ages and both sexes. For haemoglobin results below 9.9 g dl^−1^, the PPV was 2.0% (CI 1.7, 2.3) and for 10.0–12.9 g dl^−1^, the PPV was 0.3% (0.2, 0.3).

## DISCUSSION

This is the first study large enough to estimate the risk of colorectal cancer across different ages, sexes, and levels of haemoglobin. It confirms a strong association between anaemia and cancer, with the risk rising as the haemoglobin falls; it also confirms iron deficiency as an independent predictor of cancer. The results can be used to guide individual doctors in deciding whether to refer patients with an abnormal haemoglobin result; they can also be used to improve current guidelines.

### Strengths and weaknesses

The study is large, with haemoglobin results from 13 757 primary care patients, 3193 of whom were to be diagnosed with colorectal cancer. Most of these results had been processed automatically, reducing the chance of inputting errors. We could not confirm the diagnoses of cancer, as histology results are not routinely logged on GPs’ computer systems; nevertheless, it is unlikely that such a serious diagnosis would be entered erroneously. Similarly, we could not identify alternative causes for anaemia, microcytosis, or a low ferritin.

A higher proportion of cases than controls had had their haemoglobin measured. We accommodated this within our calculation of PPVs, by using the national incidence figures for our prior odds, using the following equation: 
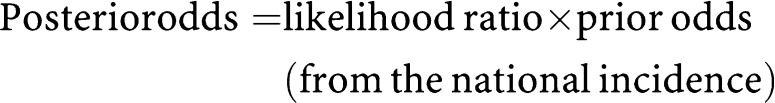
 One possibility is that differential rates of haemoglobin estimation made this calculation unreliable. The alternative was to study only those patients in whom a blood result was available. The difficulty with this approach is that the national incidence figures would not then pertain, as the simple fact of requesting testing of the haemoglobin selects a group in whom the risk of cancer is now twice the national risk *–* in that something, probably symptoms, triggered estimation of the haemoglobin. 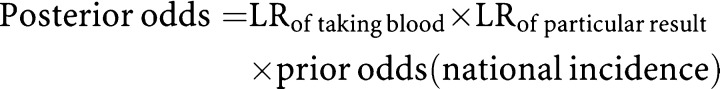


In this equation, the LR _of taking blood_ was approximately 2, and the LRs _of particular result_ were approximately half the LRs we quote in this paper. Thus, the effects cancel each other out. However, by avoiding any bias from the different levels of testing between cases and controls, we had to make another assumption, namely that all untested patients had normal haemoglobin values. This is unlikely to be true, with untested cases probably more likely to have undiagnosed anaemia than untested controls. If so, our PPVs are slight underestimates.

We also studied only one feature – anaemia. Some patients might have had additional symptoms making a referral decision easy (or, conversely, might have an alternative explanation for the anaemia rendering referral unnecessary). It is likely that patients with accompanying symptoms would have higher PPVs (and conversely those with isolated anaemia have lower PPVs) than we have calculated. This may be less important than it appears: a common clinical problem in primary care (or any setting offering care to unselected patients) is of an unexpected finding of anaemia when colorectal cancer is not being considered, and the clinician has to decide the likelihood of cancer. Furthermore, current guidance uses only one variable; anaemia. To examine this guidance critically, we have to use the same single variable. Similarly, we studied only one diagnosis – colorectal cancer. Investigation of anaemia may be appropriate for other diagnoses, such as coeliac disease (Cannings-John *et al*, 2007). This is incorporated into the BSG guidance. However, this does not change the value of our figures with respect to cancer, and their impact on cancer guidance.

The overall PPVs in this study are lower than those in the two studies performed in the referred population (which identified colorectal cancer in 6.3 and 7.4%) (Logan *et al*, 2002; James *et al*, 2005). However, they are similar to figures in one previous primary care study of 0.97% for a haemoglobin 10.0–12.9 g dl^−1^ and 2.3% for haemoglobin <10.0 g dl^−1^ in patients over 40 years (Hamilton *et al*, 2005). This illustrates once again the higher risk in the referred population compared to the primary care population as a whole, emphasising the need to use primary care data to guide primary care decisions (Hamilton and Sharp, 2004).

### Comparison with current guidance

One major problem with current guidance is that no threshold figure has been published for the risk of cancer warranting urgent investigation. Patients, doctors, and commissioners will each have a viewpoint on this. The current cancer detection rate in UK rapid investigation clinics is 6–11%; however, these clinics identify only around a third of colorectal cancers (Rai and Kelly, 2007). This reflects an important but awkward fact – less than half of the patients with colorectal cancer have a high-risk symptom, such as rectal bleeding, with its PPV for cancer being 2.4–5.7% (Ellis and Thompson, 2005; Hamilton *et al*, 2005; du Toit *et al*, 2006). The majority have softer symptoms, such as abdominal pain, constipation, or diarrhoea, each with risks of cancer of around 1% (Hamilton *et al*, 2005). If a higher proportion of symptomatic cancers is to be detected through rapid investigation clinics, then a lower threshold risk – perhaps as low as 2% – has to be accepted. We consider most patients would wish to have urgent investigation with a risk of this magnitude. This is tacitly accepted by current guidance, in which referral for persistent diarrhoea is advised, even though the risk of cancer after two primary care attendances with diarrhoea is still quite low, at around 1.5% (Hamilton *et al*, 2005).

The current recommendations for anaemia (which require the presence of iron deficiency as well as anaemia) had PPVs of 13.3% for men and 7.7% for women. This clear sex difference remained for haemoglobin values above the current referral threshold. Positive predictive values in men were above 4% for all haemoglobin values up to 12.0 g dl^−1^, whereas women's PPVs were generally lower. This reflects the higher overall incidence of colorectal cancer in men, together with the lower mean haemoglobin in all ages of women, not just in those who are premenopausal (Guralnik *et al*, 2004). Nonetheless, it can be argued that in patients with iron deficiency, any degree of anaemia up to 12 g dl^−1^ warrants urgent investigation (as the BSG guidance suggests, albeit without stating urgent investigation is warranted). Simpler guidance would be to ignore features of iron deficiency entirely and recommend urgent referral of patients over 60 with unexplained anaemia on the haemoglobin value alone. If this were done, the current threshold figures of 11 g dl^−1^ for men and 10 g dl^−1^ for women appear reasonable, with almost all PPVs above 2%, and most well above this figure.

This study provides an evidence base for investigation of anaemia detected in primary care. As always, there is a trade-off between identifying as many cancers as possible (sensitivity) and keeping the number of false-positives reasonable (specificity). Colorectal cancer generally presents with low-risk symptoms, so if earlier symptomatic diagnosis is to be achieved, lower specificity cannot be avoided. The current recommendations will miss some cancers, as the threshold haemoglobin figures are low. We recommend that the requirement for iron deficiency is removed from current guidance, while the same threshold levels of haemoglobin are retained.

## Figures and Tables

**Table 1:** Age and sex of cases and controls, in the whole cohort and in those with a haemoglobin estimation

	**In whole cohort**				**With a haemoglobin estimation**	
	**Number of cases**		**Number of controls**		**Number of cases**	**Number of controls**
**Age**	**Total (%)**	**Men (%)**	**Total (%)**	**Men (%)**	**Total (%)**	**Total (%)**
30–59	1179 (18)	648 (55)	8254 (18)	4537 (55)	489 (41)	1158 (14)
60–69	1600 (25)	922 (58)	11 204 (25)	6460 (58)	748 (47)	2215 (20)
70–79	2127 (33)	1180 (55)	14 901 (33)	8272 (56)	1085 (51)	3823 (26)
80+	1536 (24)	671 (44)	10 707 (24)	4659 (44)	861 (56)	3318 (31)
Total	6442 (100)	3421 (53)	45 066 (100)	23 928 (53)	3183 (49)	10 514 (23)

**Table 2:** Haemoglobin results in cases and controls, by sex

	**Men**			**Women**		
**Haemoglobin band (g dl^−1^)**	**Number (%) in cases**	**Number (%) in controls**	**Positive likelihood ratio (CI)**	**Number (%) in cases**	**Number (%) in controls**	**Positive likelihood ratio (CI)**
<9.0	178 (5.2)	47 (0.2)	27 (19, 36)	221 (7.3)	36 (0.2)	43 (30, 61)
9.0–9.9	118 (3.5)	49 (0.2)	17 (12, 23)	146 (4.8)	85 (0.4)	12 (9.2, 16)
10.0–10.9	129 (3.8)	131 (0.6)	6.9 (5.4, 8.8)	226 (7.5)	225 (1.1)	7.0 (5.9, 8.4)
11.0–11.9	171 (5.0)	293 (1.2)	4.1 (3.4, 4.9)	238 (7.9)	616 (2.9)	2.7 (2.3, 3.1)
12.0–12.9	203 (5.9)	572 (2.4)	2.5 (2.1, 2.9)	289 (9.6)	1337 (6.3)	1.5 (1.3, 1.7)
>12.9	805 (23.5)	4131 (17.3)	1.4 (1.3, 1.5)	459 (15.2)	2992 (14.2)	1.1 (0.98, 1.2)
Unmeasured	1817 (53.1)	18 705 (78.2)		1442 (47.7)	15 847 (75.0)	
Total	3421	23 928		3021	21 138	

**Table 3:**
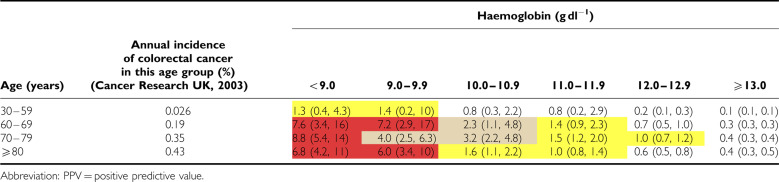
PPV for colorectal cancer of haemoglobin result taken in primary care, in men, by age, expressed as a percentage (with 95% CI)

**Table 4:**
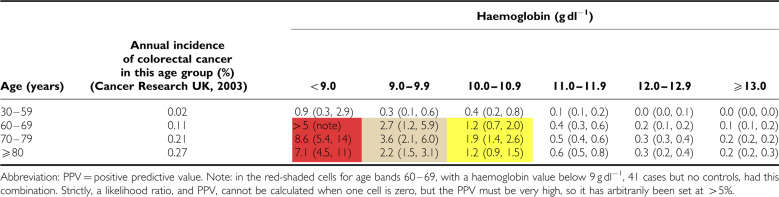
PPV for colorectal cancer of haemoglobin result taken in primary care, in women, by age, expressed as a percentage (with 95% CI)

**Table 5:**
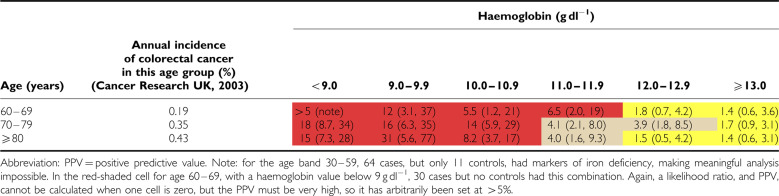
PPV for colorectal cancer of haemoglobin result accompanied by indicators of iron deficiency, in men, by age, expressed as a percentage (with 95% CI)

**Table 6:**
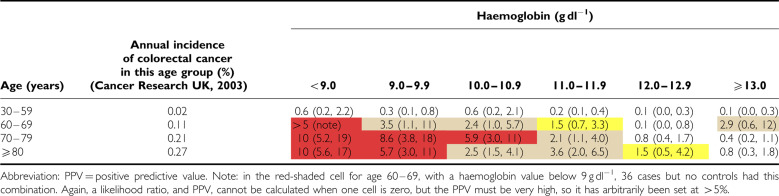
PPV for colorectal cancer of haemoglobin result accompanied by indicators of iron deficiency, in women, by age, expressed as a percentage (with 95% CI)
